# Improvement of the conjugation transfer of *N. gerenzanensis* based on the synergistic effect of quorum sensing and antibiotic interference

**DOI:** 10.1186/s13568-023-01641-9

**Published:** 2023-11-25

**Authors:** Shi shi, Yutong Cheng, Shuai Wang, Xiangmei Zhang, Fubo Han, Xiaojing Li, Huijun Dong

**Affiliations:** School of Pharmaceutical Sciences, Liaocheng University, Liaocheng, 252000 People’s Republic of China

**Keywords:** Glycopeptide antibiotic, Intergeneric conjugation, *N. gerenzanensis*, Quorum sensing, Rare actinomycetes

## Abstract

*Nonomuraea gerenzanensis* (*N. gerenzanensis*) is known for its ability to biosynthesize A40926, the precursor of the glycopeptide antibiotic (GPA) Dalbavancin. However, challenges and uncertainties related to the genetic manipulation of the rare actinomycetes remain. In order to improve the conjugation transfer of *N. gerenzanensis*, the crucial factors affecting conjugal transfer were evaluated, including agar medium, mycelial state, donor-recipient ratio, magnesium ion concentration, and antibiotic coverage time firstly. Additionally, γ-butyrolactone (GBL) for quorum sensing (QS) and antibiotics targeting bacterial walls were applied to evaluate their effects on conjugation transfer. As a result, the optimal conditions of 5%TSB of liquid medium, 24 h of the period time, V0.1 of agar medium, 30 mM of magnesium ion, the ratio 10:1 of donor-to-recipient, and 27 h of the overlaying time of antibiotic were determined. Furthermore, the results showed that autoinducer GBL and GPA teicoplanin had a synergetic effect on the conjugation transfer of *N. gerenzanensis* at a working concentration of 60 µM and 0.5 µg mL^−1^, respectively. The highest conjugation efficiency could reach about 1.3 depending on the optimal process conditions and the interference of QS and antibiotics.

**Supplementary Information:**

The online version contains supplementary material available at 10.1186/s13568-023-01641-9.

## Introduction

Rare actinomycetes, namely non-*Streptomycete* actinomycetes, are an essential source of medicinally active secondary metabolites (Al-Fadhli et al. 2022; Ding et al. 2019; Subramani and Sipkema 2019). A40926 is the precursor of the GPA dalbavancin, which is biosynthesized by rare actinomycete *N. gerenzanensis* ATCC 39727 (Dalmastri et al. 2016). The whole *N. gerenzanensis* genomic DNA and the biosynthetic gene cluster (BGC) for A40926 were analyzed and extensively verified (Lo Grasso et al. 2015; Sosio and Donadio 2006; Sosio et al. 2003). In order to conduct a comprehensive and thorough study of this rare actinomycete at the molecular level, an effective genetic transfer system is needed.

So far, routine genetic manipulation of Streptomyces is intergeneric conjugation (Gao and Smith 2021; Pettis 2018; Zhang et al. 2019). For *N. gerenzanensis*, the first reported gene transfer system was developed with intergeneric conjugation (Stinchi et al. 2003). Similarly, Marcone reported two gene transfer systems for *N. gerenzanensis* including protoplast transformation and intergeneric conjugation with the site-specific integrative plasmid pSET152 and cosmid (Marcone et al. 2010a). However, the conjugation efficiency involved in these studies was not high, suggesting that fine optimization of conjugation transfer was necessary for rare actinomycetes. In this study, we have entirely improved the conjugation transfer procedure of *N. gerenzanensis*.

As in advanced organisms, bacteria can communicate on information provided by chemical signal molecules, such as acyl-homoserine lactone (AHL) of Gram-negative bacteria, oligopeptide of Gram-positive bacteria, and GBL of Streptomyces (Safari et al. 2014; Waters and Bassler 2005; Wu et al. 2021). This communication is called QS, enabling bacteria to monitor environmental changes and synchronize cell activity. Recent studies have demonstrated that the horizontal transfer of conjugation-mediated resistant plasmids strongly correlates to QS (Banderas et al. 2020; Ramsay et al. 2022; van Gestel et al. 2021). In addition, it has also been found that subinhibitory concentration antibiotics can promote conjugation transfer (Jutkina et al. 2018, 2016; Shun-Mei et al. 2018).

Therefore we attempted to apply the principle of QS and antibiotics to optimize the conjugation transfer of rare actinomycetes. In this research, the effects of autoinducer GBL and four antibiotics (ampicillin, cephalosporin, vancomycin, and teicoplanin) on the growth and the conjugation efficiency of *N. gerenzanensis* were studied*.*

## Materials and methods

### Bacterial strains, plasmids, media, and growth conditions

The bacterial strains and plasmids used in this work are presented in Additional file 1: Table S1. *N. gerenzanensis* ATCC39727 was propagated on Mannitol-Soy (MS) agar (Buttner et al. 2000) and VSP liquid medium (Marcone et al. 2010b)*.* The mycelium of *N. gerenzanensis,* which grew at 28°C in VSP medium for two days, was collected and stored in 20% glycerol at -80 °C. Routinely, *E. coli* were grown at 37°C in Luria broth (LB) medium with shaking (220 rpm) or on LB agar, supplemented with appropriate antibiotics as required. Mycelia of *N. gerenzanensis* were obtained from four different liquid media, including R3(Lo Grasso et al. 2015), VSP, YEME (Buttner et al. 2000), and tryptone soya broth (TSB). Nine solid media BTT(Sosio et al. 2003), MS, YMG(Rocha et al. 2018), R2YE(Chouayekh et al. 2007), ISP2(Ha et al. 2008), ISP3(Ha et al. 2008), OMY(Peano et al. 2012), YS (D-glucose 4 g L^−1^, yeast extract 4 g L^−1^, malt extract 10 g L^−1^, CaCl_2_ 2 g L^−1^, agar 20 g L^−1^, pH7.3), and TSBY(Buttner et al. 2000) were applied to observe the spore formation. Four solid media MS, V0.1(Marcone et al. 2010b), TSBY, and R3 were used as regenerative media for *N. gerenzanensis* exconjugant. The pH for both liquid and solid media was adjusted to 7.2.

### DNA manipulations

Isolation of genomic and plasmid DNA, DNA ligation, and other DNA manipulation were conducted by the standard protocols or guidelines of the corresponding commercial kits. The tool enzymes for gene manipulation were purchased from Nanjing Vazyme Biotechnology Co., Ltd. All chemical reagents used in this study are all molecular biology grade and purchased from Sangon Bioengineering (Shanghai) Co., Ltd, and Shanghai Aladdin Biochemical Technology Co., Ltd.

### Construction of donor cells

To detect the conjugator of *N. gerenzanensis*, the *egfp* expression element driven by the constitutive promoter *ermE* was synthesized, digested with *Nde*I and *BamH*I, and inserted into plasmid pIJ8660 treated with the same enzymes to obtain the recombinant plasmid (Additional file 1: Figure S1). The plasmid was transformed into the competent cells of *E. coli* ET12567 by the calcium chloride method. Further, ET12567 strains were inoculated to 30 mL of LB (containing 50 mg L^−1^ kanamycin, 25 mg L^−1^ chloromycetin, and 50 mg L^−1^ apramycin) in a 50-mL shaker flask and incubated at 37°C and 220 rpm for 2–3 h to OD_600_ of 0.2–0.3 at the cell titer of 1 × 10^6^–7 × 10^6^ cfu mL^−1^. *E. coli* cells were collected at 2653 × *g* and washed twice with antibiotic-free LB to remove antibiotics. *E. coli* cells were resuspended in 1.0 mL LB medium and used as donor cells.

### Intergeneric conjugation

To prepare the *N. gerenzanensis* mycelia, a single colony was transferred and cultivated in 25 mL different liquid medium in a 250-mL shake flask at 28°C and 220 rpm for 48 h. Then 2 mL of seed culture was inoculated to 50 mL of the different liquid medium in a 500-mL shake flask and continuously cultivated at 28°C and 220 rpm for 24 h. At this stage, the number of mycelial cells was about 2 × 10^6^–5 × 10^6^ cfu mL^−1^. The mycelia from 1 mL culture were collected at 2653 × *g* for 5 min, treated by GPA teicoplanin for the appropriate time, and then washed with fresh sterile LB twice. Finally, the mycelia were resuspended in 1.0 mL of LB and used as recipient cells.

The *E. coli* donor cells were transferred into the resuspended mycelia to briefly mix the homogeneity with the pipette according to the proper donor-recipient ratio. The mixture was spread on the regeneration plates (containing GBL, teicoplanin, and MgCl_2_ with appropriate concentration). After incubating at 28℃ for 21–24 h, each plate was flooded with 1 mL sterile water containing1 mg nalidixic acid and 1 mg apramycin. The plates were further incubated at 28℃ until the exconjugants appeared. Isolated clones were spread on new patches containing 50 μg mL^−1^ apramycin for confirmation of exconjugants. GBL and antibiotics used in this study are purchased from Shanghai Aladdin Biochemical Technology Co., Ltd.

### Scanning electron microscopy (SEM) analysis of mycelia

First, the mycelium of *N. gerenzanensis* was treated with a certain concentration of teicoplanin, and then washed twice with 0.1 M phosphate-buffered saline (PBS, pH 7.2) to remove the residual teicoplanin. Furthermore, the mycelia were fixed by 2.5% glutaraldehyde solution for 12 h and washed three times with 0.1 M PBS, then dehydrated with graded ethanol (50–100%) and replaced by pure acetone in order, and finally lyophilized to obtain dried mycelium. Secondly, the dried mycelium was sprayed with gold by ion sputtering apparatus for 20 s and observed by SEM (FlexSEM 1000 II, Hitachi, Japan).

### PCR confirmation for exconjugants

The exconjugant genomic DNA was extracted and then confirmed by the eGFP forward primer (5´-atggtgagcaagggcgaggagctgt-3´) and the reverse prime (5´-ttacttgtacagctcgtccatgccg-3´). They were designed to amplify *egfp* and verify whether the gene was integrated into the exconjugant genome. The PCR product size of *egfp* was 720 bp. The amplifier was sequenced and compared to the sequence in GenBank.

### Mycelial observation

In order to verify the integration of the *egfp* expression cassette in the conjugants, EGFP fluorescence was observed by fluorescence microscope. In detail, the recombinant strains were cultivated in a VSP medium with five glass beads (*Φ* 5 mm) at 30°C for 72 h. Then the mycelia were collected by centrifugation at 2653 × *g* for 10 min and washed three times with 0.85% NaCl solution. Subsequently, the mycelial resuspension was diluted to 0.5 of optical density at 505 nm with the same saline solution and observed by a Nicon Ti-s fluorescence microscopy with 40 objective.

## Results

### Investigation of *N. gerenzanensis* sporulation

Usually, the aerial hypha's differentiated spores are considered the ideal receipt cells for the conjugation transfer of filamentous microorganisms. However, mycelium was selected as the recipient cells in almost all published studies involving the genetic manipulation of *N. gerenzanensis* (Lo Grasso et al. 2015; Marcone et al. 2014; Stinchi et al. 2003). These studies appear to suggest that it is hard for this rare actinomycete to differentiate spores. To check the sporulation of *N. gerenzanensis*, we investigated the growth of *N. gerenzanensis* on nine agar media commonly used for Streptomyces. As shown in Additional file 1: Figure S2, the spores were not observed on any experimental agar medium. As a result, mycelium as recipient cell should be the unique option for the conjugation transfer of *N. gerenzanensis*. Moreover, it is interesting that *N. gerenzanensis* presents apparent morphology and color variations in the different media (Additional file 1: Figure S2), which reveals that *N. gerenzanensis* has plenty of secondary metabolites.

### Sensitivity of *N. gerenzanensis* to antibiotics

In microbial pathogens, plasmid-mediated conjugation transfer is one of the primary causes of bacterial resistance to antibiotics (de Nies et al. 2022; Jamieson-Lane and Blasius 2022; Nohejl et al. 2022). In general, antibiotics targeting bacterial walls or membranes are more likely to cause drug resistance, which may be related to their ability to change the permeability of the cell barrier. Therefore, we infer that antibiotics such as glycopeptides or beta-lactams may affect conjugation transfer. Cephalosporins, ampicillin, vancomycin, and teicoplanin were chosen to assess their inhibition of *N. gerenzanensis*. As shown in Table 1 and Additional file 1: Figure S3, ampicillin and cephalosporin did not inhibit the growth of *N. gerenzanensis* in the concentration range of 1 μg mL^−1^ to 80 μg mL^−1^. In contrast, vancomycin and teicoplanin showed certain inhibitory effects. In particular, teicoplanin could inhibit the growth of *N. gerenzanensis* when its concentration increased to 2.5 μg mL^−1^. Moreover, the sensitivity of *E. coli* ET12567 to teicoplanin was assessed, and the result showed that the antibiotic had no inhibition on *E. coli* (Additional file 1: Figure S4).

**Table 1:** Sensitivity of *N. gerenzanensis* to antibiotics

Antibiotics(μg mL^−1^)	1	2.5	5	10	20	40	80
Ampicillin	+ + +	+ + +	+ + +	+ + +	+ + +	+ + +	+ +
Cephalosporin	+ + +	+ + +	+ + +	+ + +	+ + +	+ + +	+ + +
Vancomycin	+ + +	+ + +	+ + +	+ + +	+ +	+ +	+
Teicoplanin	+ + +	+ +	+	−	−	−	−

+  +  + growth normal; +  + growth inhibition; + growth week;  − no growth

### Optimization of the classic conjugation transfer protocol

**Fig. 1:**
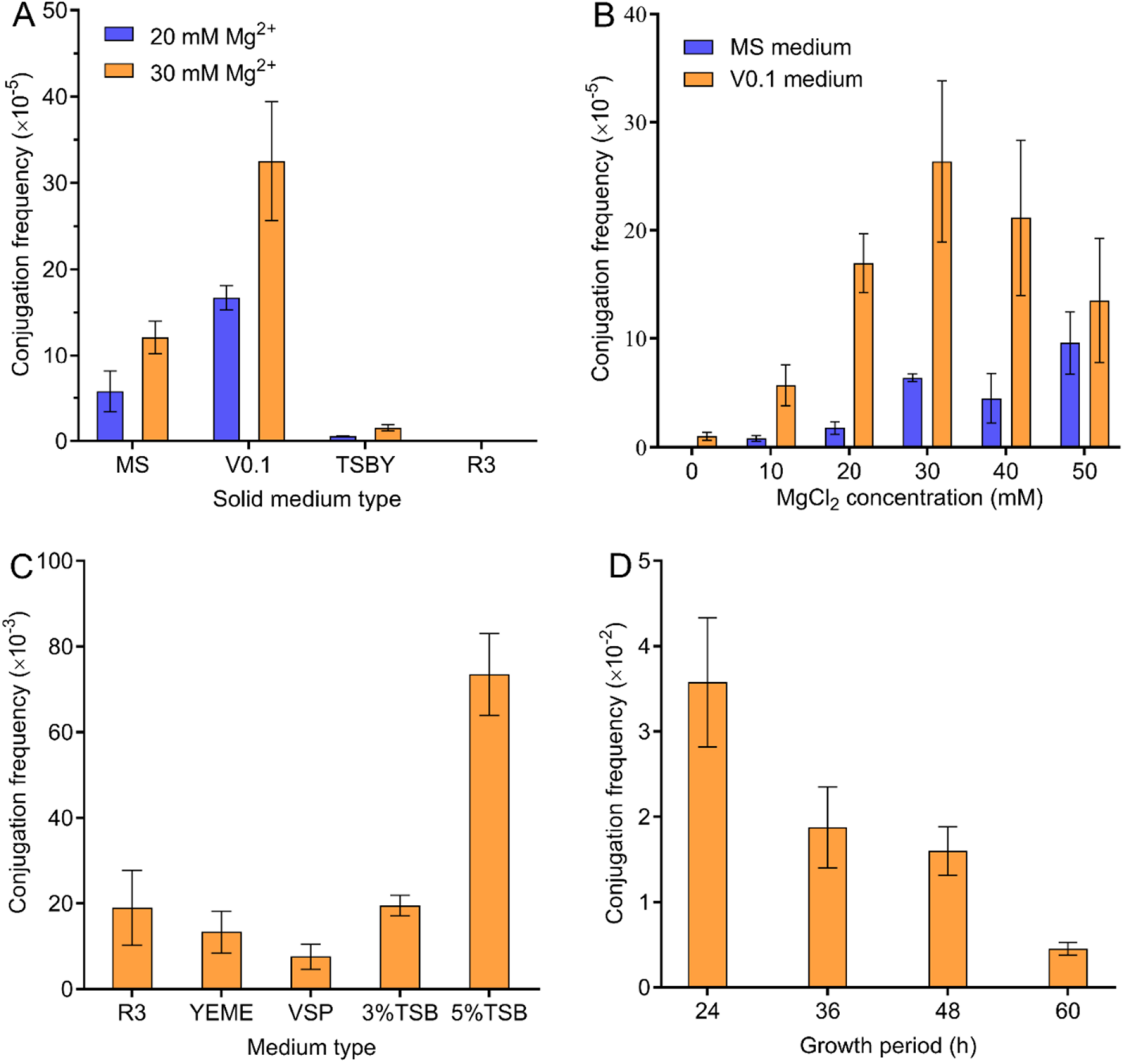
Screening and optimization of factors affecting conjugation frequency. **A**, effect of agar medium on conjugation frequency; **B**, effect of magnesium ion on conjugation frequency; **C**, effect of liquid medium on conjugation frequency; **D**, effect of cell growth state on conjugation frequency. Each point represents the mean (n = 3) ± standard deviation

The growth circumstances play an important role in the conjugation transfer between heterogeneous microorganisms. Here, four commonly used solid media MS, V0.1, TSBY, and R3 were tested to determine the optimal regenerative medium for conjugation. At the same time, 20 mM and 30 mM magnesium chloride were added to four kinds of media to evaluate their impact, respectively. The results shown in Fig. 1A demonstrated that the conjugation frequency reached the highest values of 1.67 × 10^–4^ (20 mM MgCl_2_) and 3.25 × 10^–4^ (30 mM MgCl_2_) on the V0.1 medium. In contrast, the conjugation frequency of the TSBY medium was less than 1.55 × 10^–5^ (30 mM MgCl_2_). No exconjugant colonies emerged on the R3 medium. Consequently, the V0.1 medium was determined to be the optimum conjugation medium.

The addition of magnesium has been proven to improve the conjugation transfer of actinomycetes effectively. Here, the optimal concentration of magnesium ions in the V0.1 medium was investigated, while the MS medium was used as a control. As shown in Fig. 1B and Additional file 1: Figure S5, the V0.1 medium containing 30 mM MgCl_2_ displayed the highest conjugation frequency of 2.64 × 10^–4^. In addition, the conjugation frequency decreased with the increase in concentration when the concentration of magnesium ions exceeded 30 mM. Thus, the V0.1 medium supplemented with 30 mM of MgCl_2_ was the optimal medium for the intergeneric conjugation of *N. gerenzanensis*.

Furthermore, we assessed the effect of mycelium state on the intergeneric conjugation of *N. gerenzanensis* in the different liquid media, such as R3, YEME, TSB, and VSP. The results indicated that the conjugation frequency of TSB medium (5%) was the highest, which was 7.35 × 10^–2^, while that of other media was less than 2 × 10^–2^ (Fig. 1C). Additionally, the culture time test showed that the optimal culture time was 24 h (Fig. 1D). In order to explore the reasons for the above results, we examined the growth and micromorphology of *N. gerenzanensis*. As shown in Additional file 1: Figure S6A and Fig. 1C, although the biomass in the VSP medium was significantly higher than that in other media, its conjugation frequency was the lowest. The results of micro- morphological observation suggested that the morphology of mycelium clusters in VSP, R3, and YEME medium was not conducive to conjugation, which means that a relatively small quantity of receipt cells is provided. On the contrary, the hyphae in the TSB medium were rod-shaped rather than clustered (Additional file 1: Figure S6B).

**Fig. 2:**
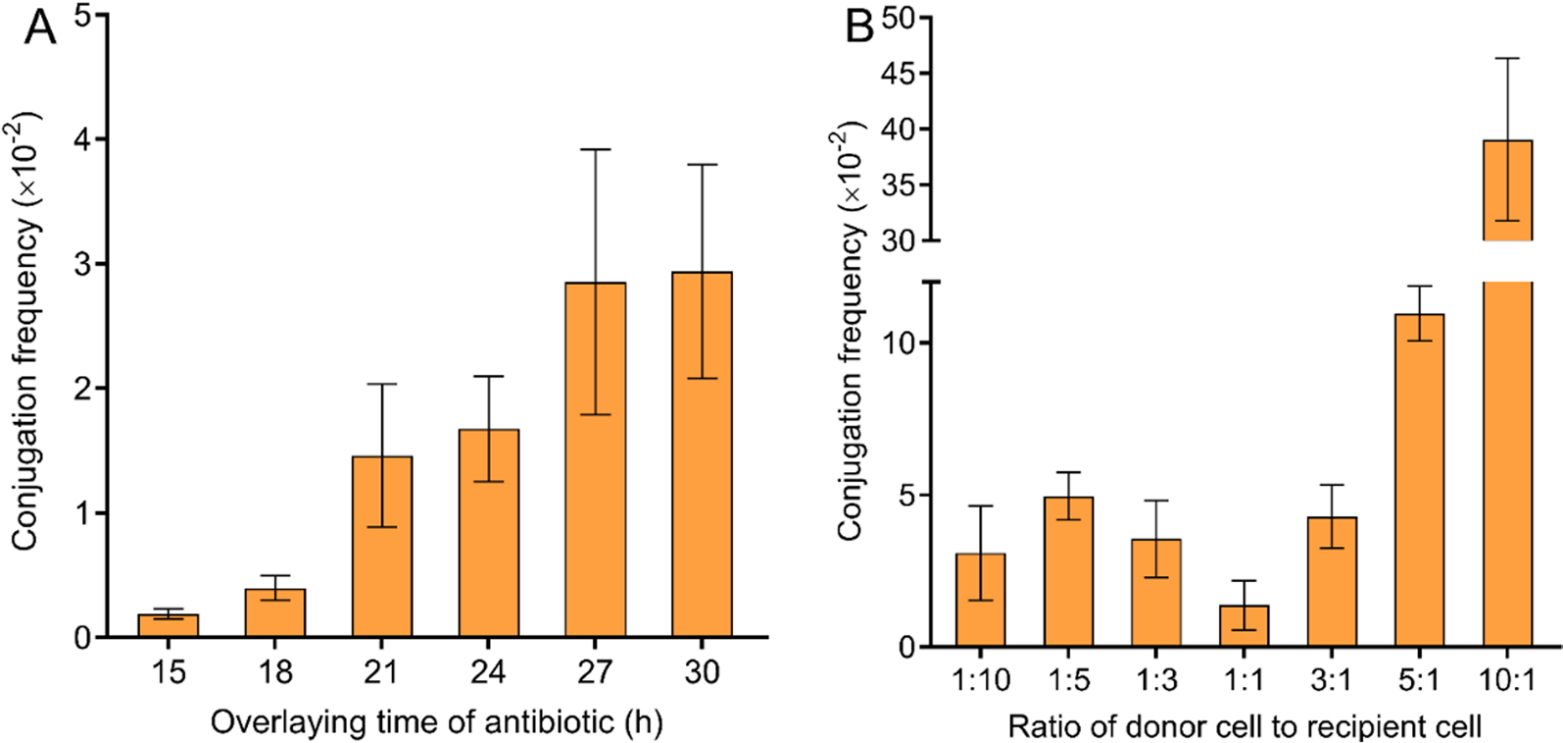
Effect of antibiotic coverage time and ratio of donor to recipient on intergeneric conjugation. **A**, antibiotic coverage time; **B**, ratio of donor to recipient. Each point represents the mean (n = 3) ± standard deviation

Finally, the antibiotic coverage time and donor-to-recipient ratio were optimized. As shown in Fig. 2A, almost no exconjugants were obtained when the coverage time was 15 h, and the conjugation frequency increased with the extension of the coverage time. The appropriate coverage time was about 27 h. The optimization result of the donor-to-recipient ratio showed that the highest conjugation frequency of 3.9 × 10^–1^ was obtained at the ratio of 10:1 (Fig. 2B).

### Effect of the autoinducer GBL on the growth and conjugation of *N. gerenzanensis*

**Fig. 3:**
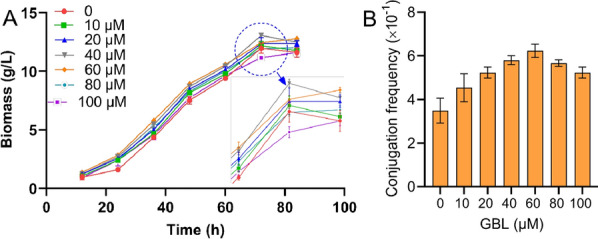
Effect of GBL on strain growth and intergeneric conjugation. **A**, growth curve of *N. gerenzanensis* in 5% TSB medium with different concentrations of GBL; **B**, effect of GBL on conjugation frequency. Each point represents the mean (n = 3) ± standard deviation

The QS mechanism exists extensively in microorganisms and is closely related to the horizontal transfer of genetic elements through conjugation transfer (Banderas et al. 2020; Breuer et al. 2018; Lin et al. 2021). In the process of QS, autoinducers are key signal molecules, such as GBL biosynthesized by streptomyces (Barreales et al. 2020; Liu et al. 2021; Xu and Yang 2019). Here, the autoinducer GBL was used to assess its effect on the growth and conjugation of *N. gerenzanensis*. As shown in Fig. 3A, the addition of GBL with different concentrations in the growth medium had little on the growth of *N. gerenzanensis*. The autoinducer GBL promoted cell growth when the concentration was less than 80 µM, but when the concentration increased to 100 µM, the cell growth was inhibited. In addition, the effect of GBL on conjugation was evaluated. The results in Fig. 3B suggested that GBL had an evident effect on the conjugation frequency. The maximum conjugation frequency increased to 0.6 when the GBL concentration was 60 µM.

### Effect of antibiotics on the conjugation of *N. gerenzanensis*

**Fig. 4:**
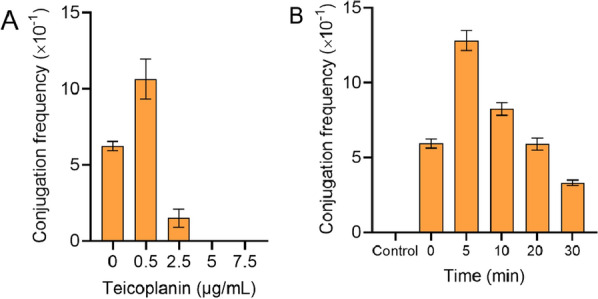
Effect of GPA teicoplanin on intergeneric conjugation. **A**, effect of teicoplanin concentration on conjugation frequency; **B**, effect of teicoplanin treatment time on conjugation frequency. Each point represents the mean (n = 3) ± standard deviation

As is well known, one of the main reasons for the emergence and spread of bacterial antibiotic resistance is the conjugational transfer of drug-resistant plasmids among closely related prokaryotic microorganisms. Some studies have confirmed that polypeptides can promote the plasmid transfer between drug-resistant bacteria (Vickerman and Mansfield 2019; Xiao et al. 2022). β-lactam and GPAs can target to destroy the wall of Gram-positive bacteria and further inhibit cell growth. Here, it is hypothesized that destroying the bacterial cell wall may promote the conjugation between bacteria. In this study, vancomycin, teicoplanin, cephalosporin, and ampicillin were selected to evaluate their effects on the growth of *N. gerenzanensis*. Vancomycin and teicoplanin could significantly inhibit the growth of *N. gerenzanensis* at concentrations of 20 µg mL^−1^ and 2.5 µg mL^−1^, respectively. However, cephalosporin and ampicillin could not inhibit the growth of *N. gerenzanensis* even if the concentration was more than 80 µg mL^−1^ (Additional file 1: Figure S3). Therefore, the GPA teicoplanin was used to evaluate its effect on the conjugation between *N. gerenzanensis* and *E. coli*. As shown in Fig. 4, teicoplanin could significantly increase the conjugation frequency at a concentration of 0.5 µg mL^−1^. However, no conjugator was found once the concentration reached 5 µg mL^−1^.

**Fig. 5:**
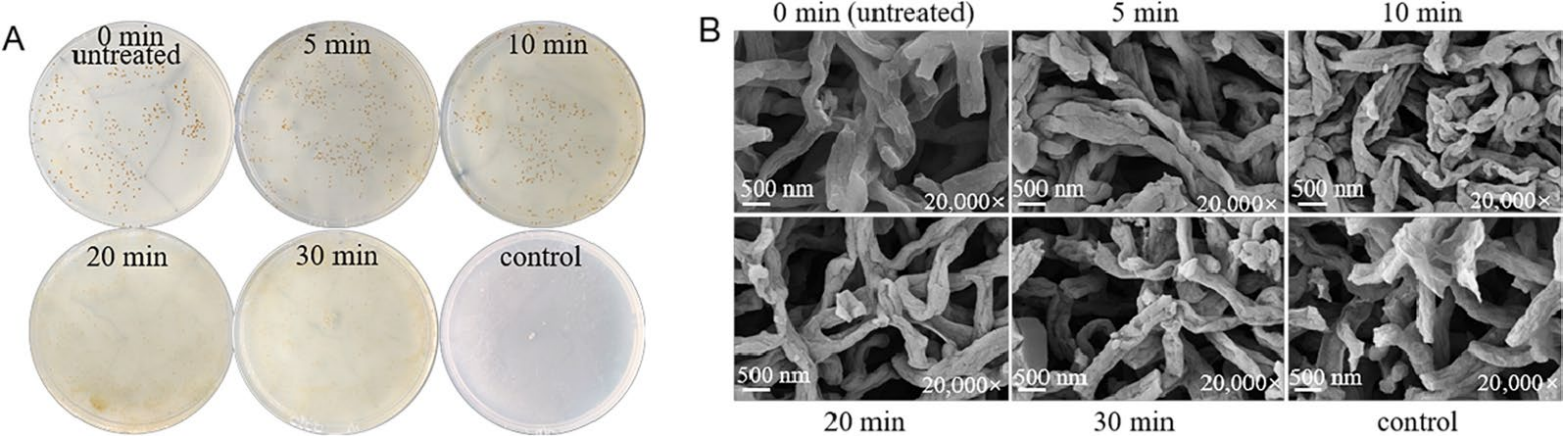
Conjugator regeneration and morphological analysis of *N. gerenzanensis* recipient cells treated with teicoplanin at different time. **A**, Conjugator regeneration plates at different time; **B**, SEM analysis of mycelium. The control sample was treated with 80 μg mL^−1^ of teicoplanin for 30 min; the other samples were treated with 0.5 μg mL^−1^ of teicoplanin for different time

Furthermore, the treatment time of teicoplanin was investigated. The results demonstrated that the best processing time was 5 min. In order to reveal the mechanism of low concentration teicoplanin promoting conjugation transfer, the mycelia treated with 0.5 µg mL^−1^ teicoplanin for the different time were observed by SEM. The results demonstrated that the wrinkles and depressions on the surface of mycelium increased with the extension of treatment time, indicating that the cell wall of mycelium changed obviously (Fig. 5B). When the concentration of teicoplanin in the control sample was 80 µg mL^−1^, the cellular debris of mycelium caused by a high concentration of teicoplanin could be observed obviously. Accordingly, no conjugator was obtained from the control sample (Fig. 5A).

### Confirmation of exconjugants.

The exconjugant was confirmed by antibiotic resistance, fluorescence observation, and amplification of the *egfp* gene. A large number of exconjugants showed resistance to the antibiotic apramycin (Additional file 1: Figure S7A), which proved that the genetic transformation was highly efficient. Subsequently, the mycelia of exconjugants cultivated in a liquid medium were collected and then observed by fluorescence microscope. As shown in Additional file 1: Figure S7B, apparent green fluorescence was observed from the mycelium. In addition, the *egfp* gene was amplified from exconjugants by PCR. Gel electrophoresis analysis showed that compared with the wild-type strain, all the selected exconjugants obtained the 720 bp PCR products of *egfp* gene (Additional file 1: Figure S7C).

## Discussion

In almost all cases of genetic manipulation for actinomycetes, conjugation transfer is considered the conventional strategy. In general, spores are ideal recipient cells to receive gene elements from donor cell *E. coli*. Nevertheless, for non-sporulating actinomycetes, mycelium was the only alternative recipient, and the conjugation efficiency was usually low (Rocha et al. 2018; Stinchi et al. 2003). This study systematically optimized the conjugation protocol of non-sporulating actinobacteria *N. gerenzanensis* to improve the conjugation frequency.

First, our research confirmed that the type of culture medium significantly affected the conjugation frequency. The V0.1 medium exhibited the highest frequency. This finding is consistent with the results of the study published by Marcone (Marcone et al. 2010a). Furthermore, we first investigated the effect of mycelial morphology on conjugation. The results showed that the short and dispersed mycelium morphology was more favorable for conjugation, depending on the type of liquid medium. Interestingly, there was no positive correlation between the biomass of the recipient strain and the conjugation frequency. In this study, the conjugation frequency of 5%TSB was the highest, but the biomass of the recipient strain was not the highest in this medium.

QS exists widely in bacteria and can affect the cell density and population behavior of bacteria, such as gene expression, secondary metabolism, and horizontal gene transfer (Banderas et al. 2020; Wu et al. 2021). Actinomycetes utilize GBL as autoinducers to regulate morphological differentiation and metabolism via QS. In this study, we applied the autoinducer GBL to the conjugation transfer process of *N. gerenzanensis*. Meanwhile, antibiotics that can destroy the cell walls of Gram-positive bacteria, including ampicillin, cephalosporin, vancomycin, and teicoplanin, are also used to study their effects on the conjugate. With the exception of teicoplanin, the other three antibiotics significantly inhibited the growth of *E. coli* ET12567 (data not shown). Therefore, the effect of teicoplanin on the conjugation was further evaluated. The results showed that GBL and teicoplanin could increase the conjugation frequency. In particular, the optimal conjugation frequency achieved with mycelia reached 1.3 × 10^0^, which was higher than the maximum value (8 × 10^–4^) reported in the previous study (Marcone et al. 2010a).

In summary, the methodology described in this work provides a highly efficient tool for conjugating *N. gerenzanensis*. It even provides a new and suitable choice for the genetic transformation of other non-sporulating actinomycetes.

### Supplementary Information

**Additional file 1: Table S1. **Strains and plasmids used in this work. **Figure S1. **Diagram of the recombinant plasmid. **Figure S2. **Sporulation examination of *N. gerenzanensis* on different agar medium. **Figure S3. **Sensitivity of *N. gerenzanensis* to different concentrations of different antibiotics. **Figure S4. **Sensitivity of *E. coli *ET12567 to different concentrations of teicoplanin. **Figure S5. **Comparison of conjugator regeneration on MS and V0.1 agar medium containing different concentrations of magnesium ions. The pictures are presented in black and white. **Figure S6. **Growth curves and microscopic check of* N. gerenzanensis* cultivated in different liquid media. A, growth curves; B, microscopic observation. Each point represents the mean (n=3) ± standard deviation. **Figure S7.** Exconjugant confirmation through the methods of antibiotic resistance, fluorescence observation and PCR. A, exconjugants grown on apramycin resistant plate. B, fluorescence observation of exconjugant mycelium. C, gel electrophoresis of amplified products of EGFP in exconjugants, M: DNA ladder, WT: wild-type strain, 1-7: seven random exconjugants.

## Data Availability

All data generated or analyzed during this study are included in this manuscript and Additional file.
