# Supplementary material for: Improvement of the conjugation transfer of N. gerenzanensis based on the synergistic effect of quorum sensing and antibiotic interference
Source: AMB Express. 2023 Nov 25;13:133. doi: 10.1186/s13568-023-01641-9 (PMC10676335; doi:10.1186/s13568-023-01641-9)
Supplement: Supplementary file 1 — Additional file 1: Table S1. Strains and plasmids used in this work. Figure S1. Diagram of the recombinant plasmid. Figure S2. Sporulation examination of N. gerenzanensis on different agar medium. Figure S3. Sensitivity of N. gerenzanensis to different concentrations of different antibiotics. Figure S4. Sensitivity of E. coli ET12567 to different concentrations of teicoplanin. Figure S5. Comparison of conjugator regeneration on MS and V0.1 agar medium containing different concentrations of magnesium ions. The pictures are presented in black and white. Figure S6. Growth curves and microscopic check of N. gerenzanensis cultivated in different liquid media. A, growth curves; B, microscopic observation. Each point represents the mean (n=3) ± standard deviation. Figure S7. Exconjugant confirmation through the methods of antibiotic resistance, fluorescence observation and PCR. A, exconjugants grown on apramycin resistant plate. B, fluorescence observation of exconjugant mycelium. C, gel electrophoresis of amplified products of EGFP in exconjugants, M: DNA ladder, WT: wild-type strain, 1-7: seven random exconjugants. [file 13568_2023_1641_MOESM1_ESM.docx]

**Supplementary information**

1. **Supplementary Tables**

**Table 1.** Strains and plasmids used in this work

| Strains and plasmids | Description | Source or reference |
| --- | --- | --- |
| *Nonomuraea gerenzanensis* | Wild-type, glycopeptide substance A40926 producer | ATCC 39727 |
| *N. gerenzanensis* EE | The genome DNA of ATCC 39727 with the exogenous *ermE* promoter and eGFP gene | This study |
| *E.coli* DH5α | Routine subcloning host | [1] |
| *E.coli* ET12567 (pUZ8002) | Cloning host (methylation defective), used for conjugative transfer of DNA and for demethylating plasmid DNA | [2] |
| ET-pIJ-eGFP | Strain ET12567 that contains pIJ8660 plasmid with promoter *ermE* and eGFP gene. Am^R^, Km^R^, Cm^R^ | This study |
| pIJ8660 | A pSET152 derivative. Am^R^ | [3] |
| pIJ8660-*ermE*p-eGFP | pIJ8660 plasmid with *ermE* promoter and eGFP gene | This study |

*Apramycin resistance (Am^R^), kanamycin resistance (Km^R^), chloramphenicol resistance (Cm^R^).

1. **Supplementary Figures**


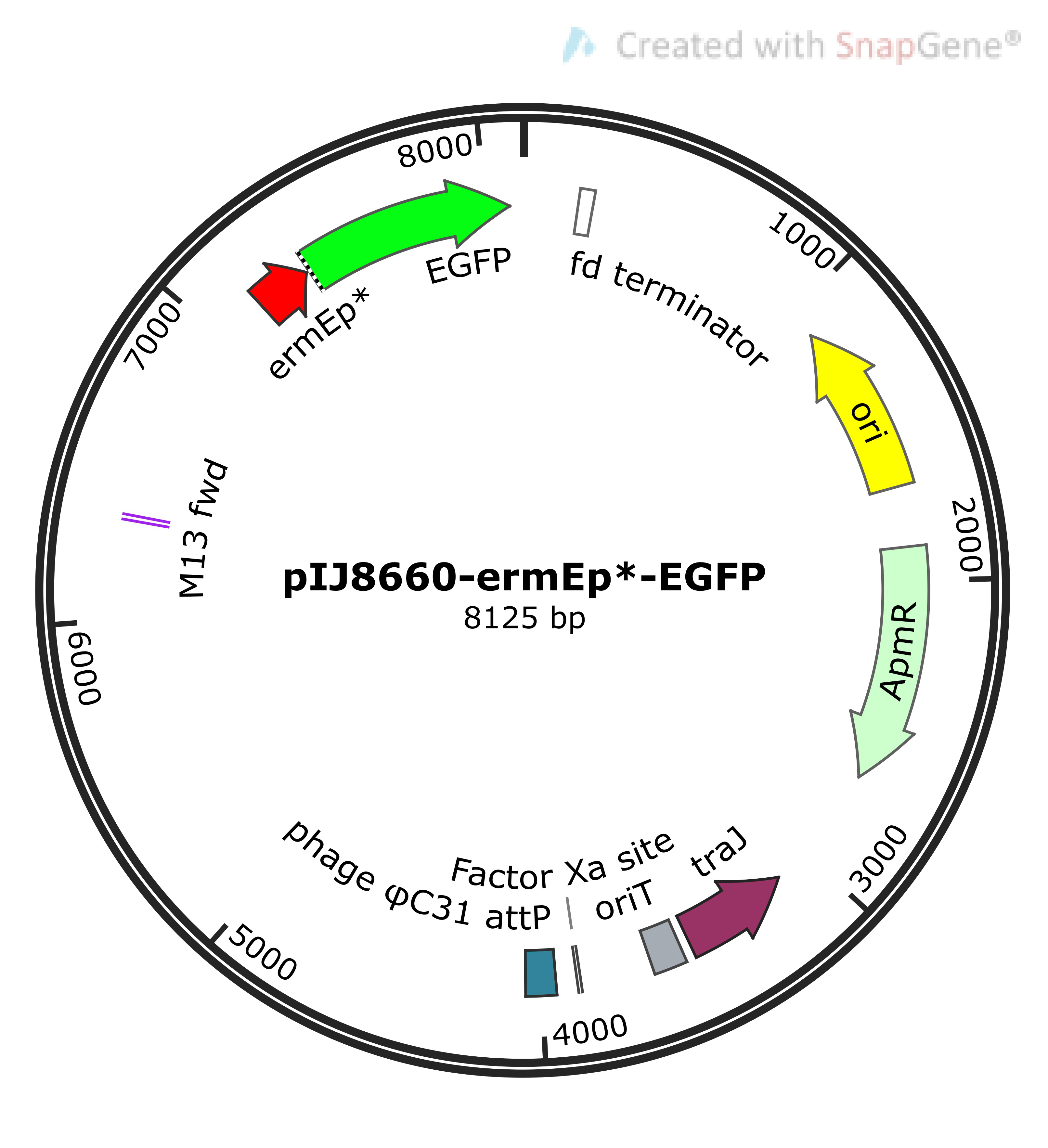


**Figure 1.** Diagram of the recombinant plasmid.


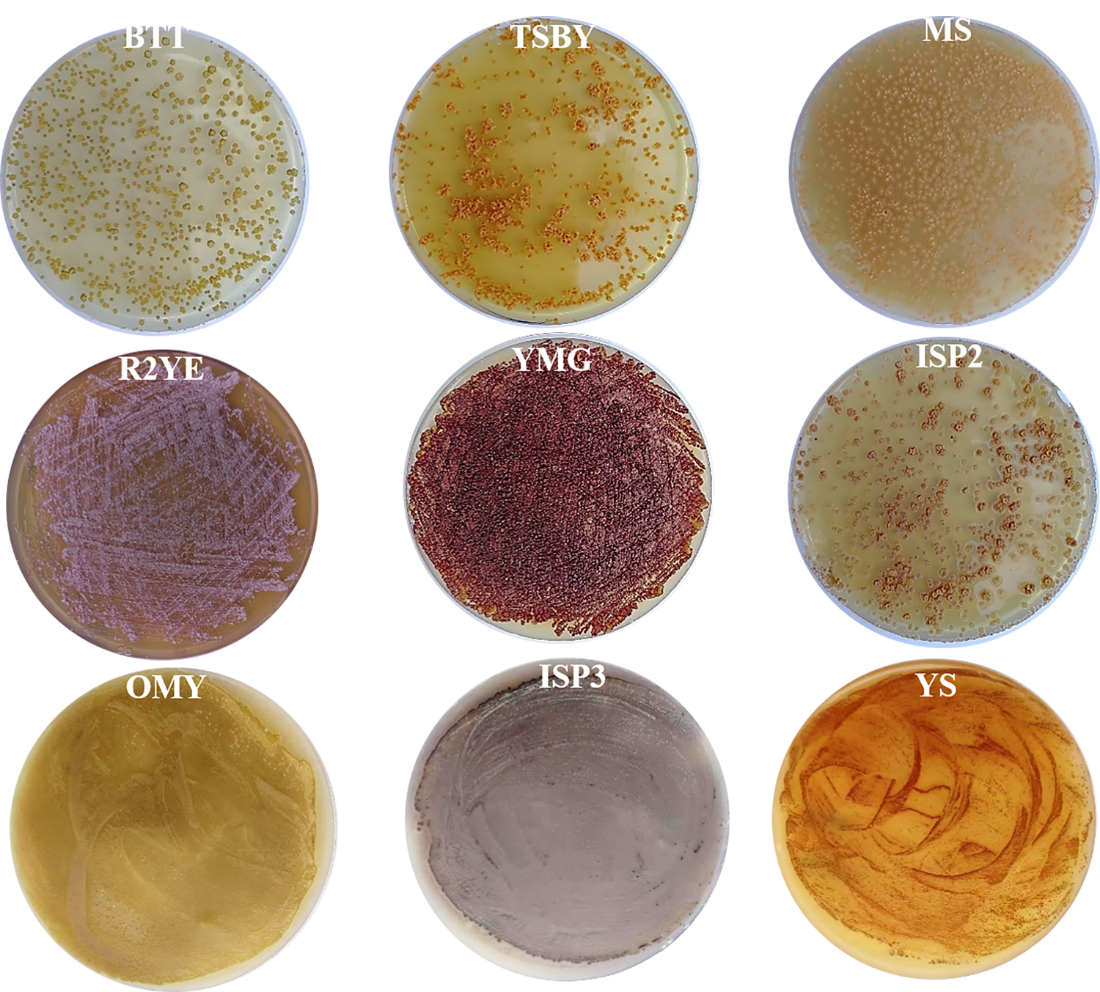


**Figure 2.** Sporulation examination of *N. gerenzanensis* on different agar medium.


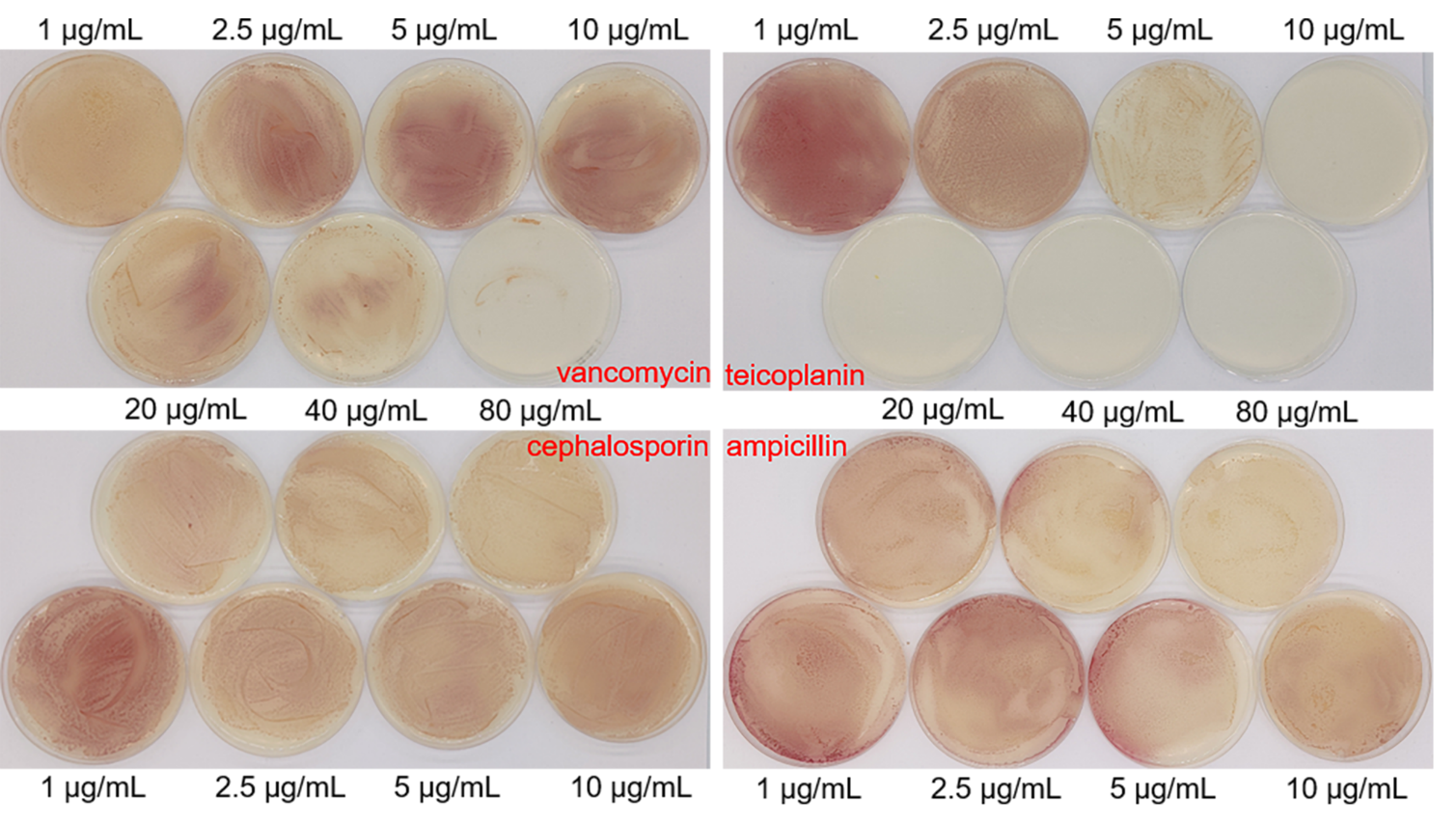


**Figure 3.** Sensitivity of *N. gerenzanensis* to different concentrations of different antibiotics.


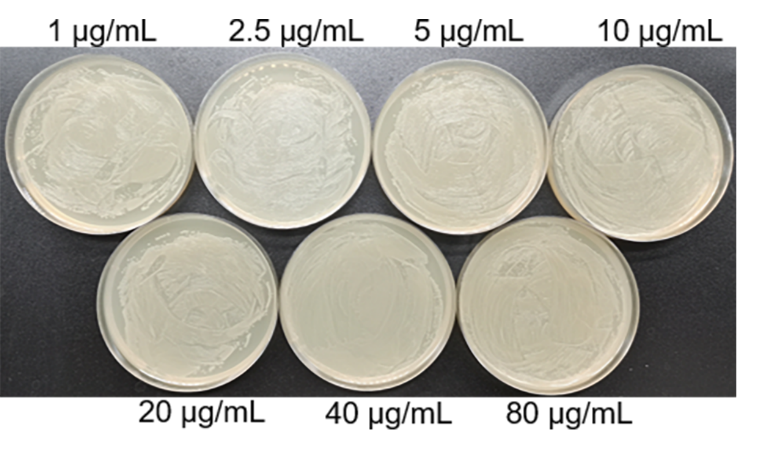


**Figure 4.** Sensitivity of *E. coli* ET12567 to different concentrations of teicoplanin.

**MS+30 mM MgCl_2_**

**MS+20 mM MgCl_2_**

**V0.1+20 mM MgCl_2_**

**V0.1+30 mM MgCl_2_**


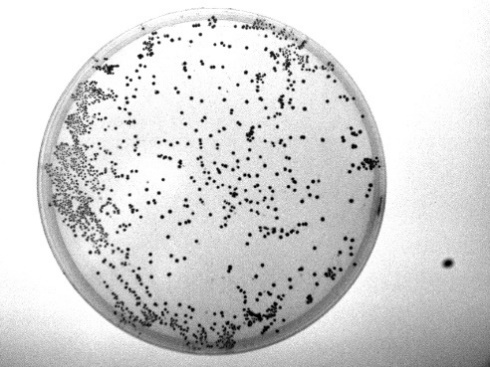

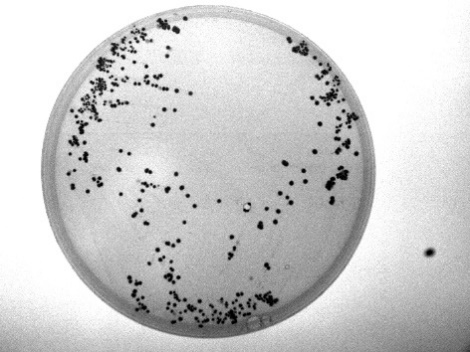

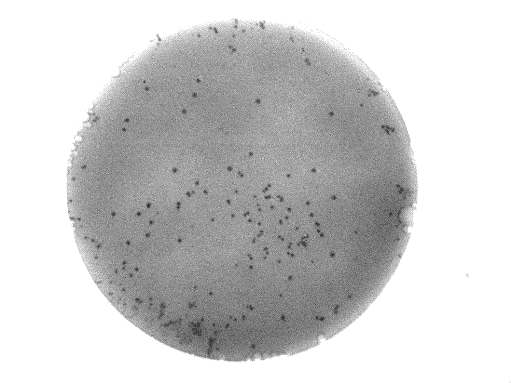

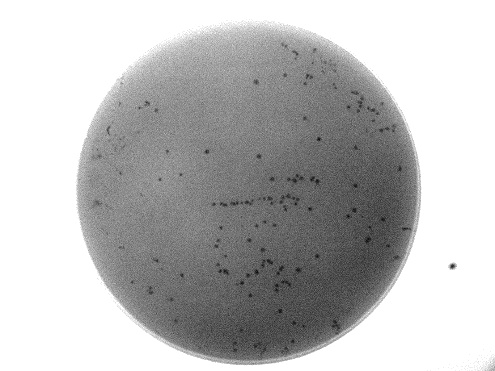


**Figure 5.** Comparison of conjugator regeneration on MS and V0.1 agar medium containing different concentrations of magnesium ions. The pictures are presented in black and white.


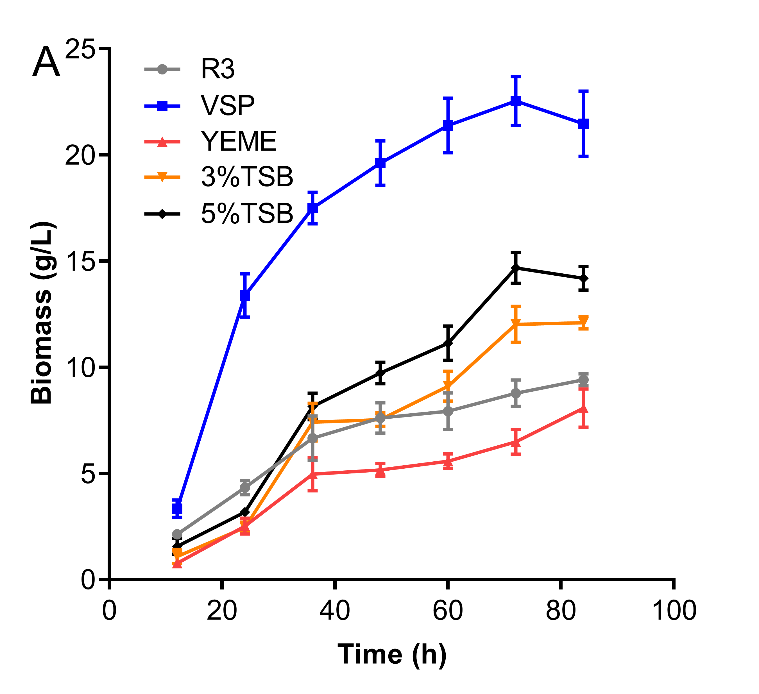

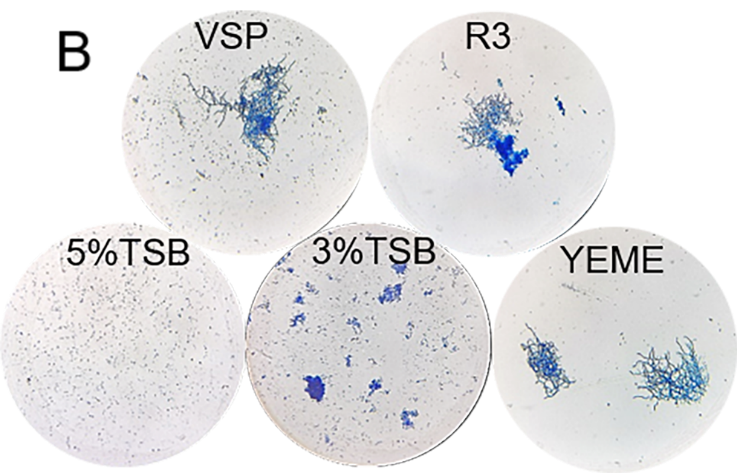


**Figure 6.** Growth curves and microscopic check of *N. gerenzanensis* cultivated in different liquid media. A, growth curves; B, microscopic observation. Each point represents the mean (n=3) ± standard deviation


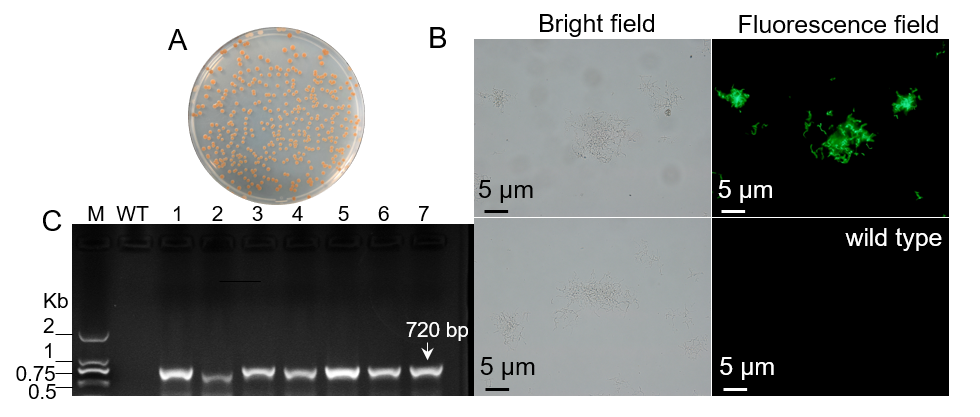


**Figure 7.** Exconjugant confirmation through the methods of antibiotic resistance, fluorescence observation and PCR. A, exconjugants grown on apramycin resistant plate. B, fluorescence observation of exconjugant mycelium. C, gel electrophoresis of amplified products of EGFP in exconjugants, M: DNA ladder, WT: wild-type strain, 1-7: seven random exconjugants.

**Reference**

1. Hanahan D (1983) Studies on transformation of *Escherichia coli* with plasmids. J Mol Biol 166 (4):557-580. doi:10.1016/s0022-2836(83)80284-8

2. MacNeil DJ, Gewain KM, Ruby CL, Dezeny G, Gibbons PH, MacNeil T (1992) Analysis of *Streptomyces avermitilis* genes required for avermectin biosynthesis utilizing a novel integration vector. Gene 111 (1):61-68. doi:10.1016/0378-1119(92)90603-m

3. Santos-Beneit F, Errington J (2017) Green fluorescent protein as a reporter for the spatial and temporal expression of actIII in *Streptomyces coelicolor*. Arch Microbiol 199 (6):875-880. doi:10.1007/s00203-017-1358-1
